# 2089. Relative patient preferences for starting daily, on-demand, and long-acting injectable HIV pre-exposure prophylaxis among US men who have sex with men, 2021-2022

**DOI:** 10.1093/ofid/ofac492.1711

**Published:** 2022-12-15

**Authors:** Travis Sanchez, S Wilson Beckham, Marissa J Hannah, O Winslow Edwards, Keith Rawlings, Alex R Rinehart, Supriya Sarkar, Patrick S Sullivan, Vani Vannappagari

**Affiliations:** Emory University, Atlanta, GA; Johns Hopkins Bloomberg School of Public Health, Baltimore, Maryland; Emory University, Atlanta, GA; Emory University, Atlanta, GA; ViiV Healthcare, Durham, North Carolina; ViiV Healthcare, Durham, North Carolina; ViiV Healthcare, Durham, North Carolina; Emory University, Atlanta, GA; ViiV Healthcare, Durham, North Carolina

## Abstract

**Background:**

Daily oral (DO) HIV pre-exposure prophylaxis (PrEP) effectively prevents HIV acquisition, but few men who have sex with men (MSM) currently use it. Newer options, such as on-demand (OD) oral and long-acting injectable (LA) PrEP may improve uptake, but little is understood about relative preferences among these options in practical start scenarios. Preferences for starting various PrEP options were examined among a US nationwide online convenience sample of MSM age 15+ collected September 2021 to February 2022.

**Methods:**

Participants reporting no prior HIV diagnosis were given brief descripitions of each PrEP option and were asked “If *[PrEP option]* were available from your local doctor and you could access it for free, would you go to your doctor in the next month to start *[PrEP option]*?” Those who said yes to multiple options were asked to rank them in order of preference. MSM currently taking DO PrEP were asked whether they would switch to OD or LA. Willingness to start LA was examined by age, race/ethnicity, insurance, and prior awareness of LA.

**Results:**

Of 5585 MSM not currently using DO PrEP, 50% (n=2805) would start at least one option with greatest preference for OD (Figure 1). Among this group, 73% (n=2060) were willing to start more than one option, with LA the most preferred option. Among the 27% (n=745) who would start only one, OD was the most preferred. Overall, 58% of DO PrEP users (n=1342/2332) would switch to either OD or LA, with LA being most preferred (Figure 2). Hispanic MSM who were not DO PrEP users were more likely to start LA compared to white MSM, and those with other/multiple health insurance were less likely to start LA compared to those on private health insurance (Table 1). Regardless of current DO PrEP use, MSM aware of LA were more likely to start it.

**Figure:**
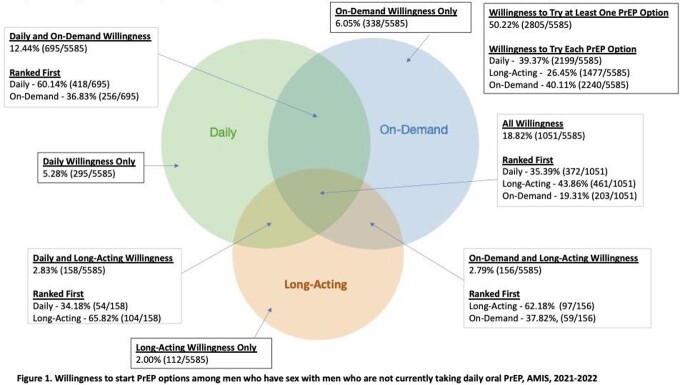

**Figure:**
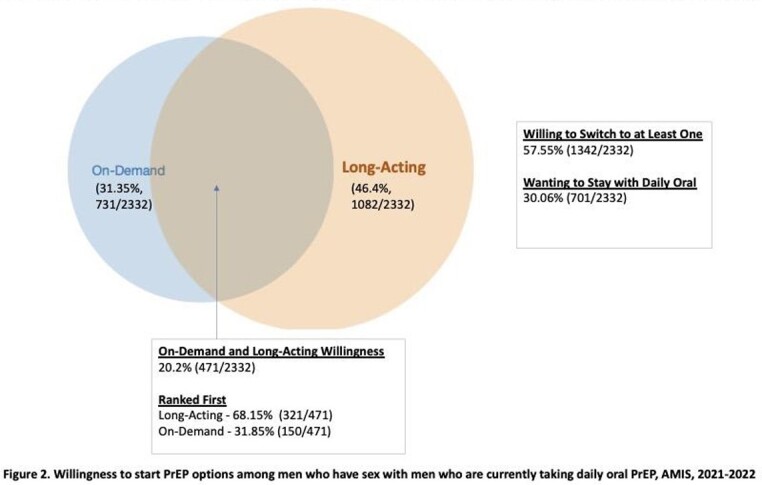

**Figure:**
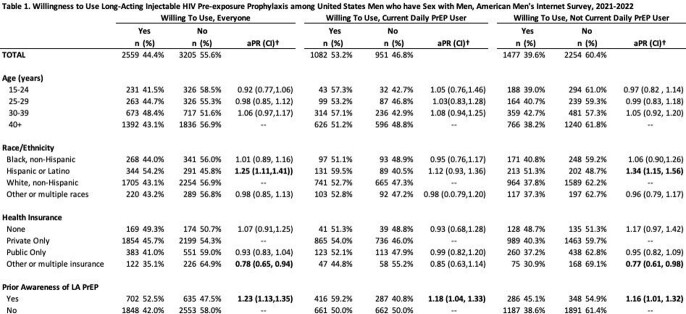

**Conclusion:**

There is substantial interest in new PrEP options. Current DO PrEP users appear to be more aware of and interested in LA than PrEP naïve. Although OD PrEP may be favored by those who are PrEP-naïve, most selected multiple options and preferred LA PrEP. Increasing awareness of LA PrEP may bolster interest in its use. These findings highlight the potential role that newer PrEP options will play in community uptake of PrEP and can also inform patient-provider decisions about which PrEP options to consider.

**Disclosures:**

**Travis Sanchez, DVM, MPH**, ViiV Healthcare: Grant/Research Support **S. Wilson Beckham, PhD, MPH, MA**, Viiv Healthcare: Advisor/Consultant **Keith Rawlings, MD**, ViiV Healthcare: Employee **Alex R. Rinehart, PhD**, ViiV Healthcare: Stocks/Bonds **Supriya Sarkar, PhD, MPH**, ViiV Healthcare: Salary|ViiV Healthcare: Stocks/Bonds **Vani Vannappagari, MBBS, MPH, PhD**, ViiV Healthcare: I am full time employee of ViiV Healthcare and receive GlaxoSmithKline stock as part of my compensation package|ViiV Healthcare: Stocks/Bonds.

